# A linear association between serum uric acid and 28-day and 90-day mortality after veno-arterial extracorporeal membrane oxygenation in patients with acute myocardial infarction complicated by cardiogenic shock

**DOI:** 10.3389/fcvm.2026.1839659

**Published:** 2026-07-27

**Authors:** Ruike Ma, Xiaoxiao Zhou, Shang Gu, Yifeng Du, Wei Zhang, Zongwei Gao

**Affiliations:** 1 Department of ECMO and Organ Support, Linyi People’s Hospital, Linyi, China; 2 Department of Breast Surgery, Linyi Cancer Hospital, Linyi, China

**Keywords:** acute myocardial infarction, cardiogenic shock, extracorporeal membrane oxygenation, mortality, uric acid

## Abstract

**Objective:**

This study aimed to delineate the relationship between serum uric acid (UA) and 28-day and 90-day mortality among patients with acute myocardial infarction complicated by cardiogenic shock (AMI-CS) receiving veno-arterial extracorporeal membrane oxygenation (VA-ECMO).

**Methods:**

In this retrospective, single-center cohort study, we consecutively enrolled 142 patients supported with VA-ECMO for AMI-CS. Baseline UA was treated as the exposure of interest, and 28-day and 90-day mortality were prespecified primary outcomes. Associations between UA and mortality were evaluated using Cox proportional-hazards models, Kaplan–Meier estimates, receiver operating characteristic curve (ROC), and restricted cubic spline (RCS) regression.

**Results:**

Among 142 patients, the median baseline UA level was 452.00 μmol/L (361.00–561.75 μmol/L), and 28-day and 90-day mortality were 33.8% and 40.8%, respectively. After adjustment for potential confounders, each 1 μmol/L increment in UA remained associated with higher hazards of 28-day mortality (HR: 1.002, 95% CI: 1.000–1.003; *P* = 0.038) and 90-day mortality (HR: 1.002, 95% CI: 1.001–1.004; *P* = 0.005). Per one standard-deviation increase in UA, the hazards of 28-day and 90-day mortality increased by 37.9% (HR: 1.379, 95% CI: 1.019–1.867; *P* = 0.038) and 49.4% (HR: 1.494, 95% CI: 1.126–1.982; *P* = 0.005), respectively. Relative to the low-UA group, the high-UA group exhibited substantially higher risks of both 28-day (HR: 2.499, 95% CI: 1.335–4.677; *P* = 0.004) and 90-day mortality (HR: 2.262, 95% CI: 1.222–4.188; *P* = 0.009). ROC analyses indicated a moderate discriminatory performance of UA for 28-day and 90-day mortality, with AUCs of 0.631 (95% CI: 0.532–0.729; *P* = 0.011) and 0.621 (95% CI: 0.527–0.714; *P* = 0.015), respectively. Kaplan–Meier curves demonstrated significant separation in cumulative mortality between UA strata, with worse survival in the high-UA group (log-rank *P* = 0.001). RCS analyses further supported a significant linear association between UA and both endpoints (*P* for nonlinearity >0.05).

**Conclusions:**

Elevated UA was associated with increased 28-day and 90-day mortality after VA-ECMO in AMI-CS. Given the single-center, retrospective, and observational design, these findings should be interpreted cautiously and require external validation before UA can be considered for clinical risk assessment.

## 1 Introduction

Cardiogenic shock (CS) remains a severe syndrome in acute cardiovascular medicine. Its pathophysiological hallmark is profound pump failure culminating in sustained tissue hypoperfusion and impaired oxygen delivery, thereby precipitating multiorgan dysfunction and death (1). Despite advances in emergent revascularization strategies, critical care techniques and mechanical circulatory support, overall outcomes remain poor, with in-hospital and short-term mortality persistently hovering around 40%–50% (2). Among CS etiologies, acute myocardial infarction complicated by cardiogenic shock (AMI-CS) is the most prevalent and is characterized by rapid clinical deterioration and marked heterogeneity, representing a continuing challenge in contemporary cardiovascular critical care. For refractory CS unresponsive to conventional therapy, veno-arterial extracorporeal membrane oxygenation (VA-ECMO) has emerged as a temporising form of mechanical support capable of rapidly restoring systemic perfusion and buying time for myocardial recovery or downstream bridging strategies (3, 4). However, several randomized trials and subsequent meta-analyses have not demonstrated a definitive survival benefit of VA-ECMO in AMI-CS, implying that although ECMO can acutely stabilize hemodynamics, longer-term outcomes may remain constrained by risk factors that are insufficiently recognized or inadequately targeted (5).

Beyond hemodynamic collapse, the CS cascade is tightly intertwined with metabolic disequilibrium, inflammatory activation and endothelial dysfunction, all of which may materially shape disease trajectories and prognosis (6). Uric acid (UA), the terminal product of purine metabolism, is widely viewed as an integrative readout of redox balance and metabolic homeostasis. Under physiological conditions, UA exerts antioxidant effects; yet in pathological states, hyperuricemia may promote cardiovascular injury via convergent mechanisms, including amplification of oxidative stress, propagation of inflammatory signalling, impairment of endothelial function and activation of the renin–angiotensin–aldosterone system (RAAS) (6, 7). Epidemiological and clinical cohort studies have consistently linked elevated UA to hypertension, coronary artery disease, heart failure (HF) and cardiovascular mortality (8–10). A large meta-analysis reported that each 1 mg/dL increase in serum UA is associated with approximately 20% higher risk of adverse cardiovascular events and 9% higher risk of all-cause mortality (10). In addition, a retrospective study of 23,475 patients suggested that UA may function as a metabolic marker for fatal AMI, with a risk threshold below the conventional diagnostic cut-off for hyperuricemia (optimal cut-off 5.70 mg/dL; fatal AMI incidence 2.6% below vs. 4.8% above; *P* < 0.0001) (11). Yet existing work has largely focused on general coronary populations or chronic HF cohorts, leaving the metabolic perturbations of AMI-CS—a severe and clinically complex state—relatively underexplored. This gap is particularly salient among AMI-CS patients supported with VA-ECMO, in whom extracorporeal circulation, ischemia–reperfusion injury, renal dysfunction and hyperinflammation may reshape UA kinetics, rendering its prognostic implications more complex. Under ECMO, conventional hemodynamic indices may be partially corrected, whereas metabolic and inflammatory signatures may provide additional prognostic information.

To date, systematic evidence delineating the relationship between UA and post-ECMO mortality risk in AMI-CS—and its dose–response form—remains limited. Against this backdrop, we conducted a single-center retrospective cohort study to interrogate the association between baseline UA and 28-day and 90-day mortality following VA-ECMO in AMI-CS, with the aim of exploring whether baseline UA is associated with short- and intermediate-term mortality in this clinically severe cohort.

## 2 Methods

### 2.1 Study population

This was a single-center retrospective cohort study. We consecutively enrolled adult patients (≥18 years) who received VA-ECMO for AMI-CS at Linyi People's Hospital between January 2021 and December 2025. Inclusion criteria were: (1) age ≥18 years; and (2) fulfilment of diagnostic criteria for AMI-CS. Exclusion criteria were: (1) no ECMO support; (2) missing baseline serum UA data; (3) patients with documented high-purine load before baseline UA assessment; or (4) loss to follow-up or refusal of follow-up. Ultimately, 142 patients were included in the final analysis. The study complied with the Declaration of Helsinki, was approved by the Ethics Committee of Linyi People's Hospital (Approval No. 202603-H-003), and since this was a retrospective study, the informed consent of all patients was waived by the Ethics Committee of Linyi People's Hospital.

### 2.2 Data collection and definitions

Clinical data were extracted from the electronic medical record system, including demographics, anthropometrics, medical history and comorbidities, severity indices, coronary angiography and intervention variables, laboratory measures, and ECMO-related treatment characteristics. Demographic variables included age, sex and smoking history; smoking was defined as any prior smoking irrespective of current smoking status (12). Anthropometric and vital-sign variables comprised body mass index (BMI = weight [kg]/height [m^2^]), systolic blood pressure (SBP), diastolic blood pressure (DBP), and heart rate (HR). Medical history and comorbidities included hypertension, diabetes, and prior myocardial infarction. Hypertension was defined as a prior diagnosis or SBP/DBP ≥ 140/90 mmHg during hospitalization (13); diabetes was defined as a prior diagnosis or fasting plasma glucose ≥7.0 mmol/L or glycated hemoglobin A1c (HbA1c) ≥6.5% during hospitalization (14). Clinical severity was characterized using the Society for Cardiovascular Angiography and Interventions (SCAI) shock staging, with inclusion restricted to stage C or higher (15). Angiography/intervention variables included number of diseased vessels, number of culprit vessels, number of stents, TIMI flow grade (0–3), and time to reperfusion. Laboratory variables included left ventricular ejection fraction (LVEF), lactate, white blood cell count (WBC), hemoglobin, platelet count (PLT), albumin, creatinine (Cr), baseline serum UA, potassium, sodium, calcium, glucose, total cholesterol, triglycerides, low-density lipoprotein cholesterol (LDL-C), high-density lipoprotein cholesterol (HDL-C), fibrinogen, high-sensitivity cardiac troponin T (hs-cTnT), creatine kinase-MB (CK-MB), and N-terminal pro-B-type natriuretic peptide (NT-proBNP). ECMO-related variables included cardiopulmonary resuscitation (CPR) prior to ECMO initiation, timing and location of ECMO cannulation, concomitant intra-aortic balloon pump (IABP) support, and durations of ECMO support, mechanical ventilation, IABP, and continuous renal replacement therapy (CRRT).

### 2.3 Measurement and stratification of UA

Serum UA was measured using a standard biochemical analyzer via the uricase–peroxidase method and reported in μmol/L. Baseline UA was defined as the available UA value measured within 24 h before VA-ECMO initiation. UA strata were assigned before subsequent ICU feeding strategies were considered, and nutritional support during VA-ECMO followed institutional critical-care protocols without routine high-purine or purine-containing supplementation. UA was analyzed as a continuous variable and also standardized as: standardized UA = (UA − mean)/standard deviation (SD). Furthermore, UA was categorized using receiver operating characteristic (ROC)-derived cut-offs based on the maximal Youden index for predicting 28-day and 90-day mortality. The corresponding apparent cut-offs were 530 μmol/L and 561 μmol/L, respectively. These cut-offs were regarded as exploratory data-driven thresholds rather than externally validated clinical decision limits. Accordingly, for descriptive and secondary categorical analyses, patients were stratified into low- and high-UA groups using the 28-day cut-off of 530 μmol/L and the 90-day cut-off of 561 μmol/L.

### 2.4 Follow-up and ascertainment of mortality

Follow-up information was obtained through review of electronic medical records (including rehospitalizations and outpatient/emergency visits) and structured telephone follow-up, capturing time of death and occurrence of death events. Survival time was defined as the interval from VA-ECMO initiation to the occurrence of an endpoint event or the end of follow-up. For descriptive analyses, patients were grouped by 28-day mortality (yes, *n* = 48; no, *n* = 94) and by 90-day mortality (yes, *n* = 58; no, *n* = 84).

### 2.5 Statistical analysis

Analyses were performed using SPSS version 26.0 and R version 4.4.3. Continuous variables were assessed for normality using the Shapiro–Wilk test. Normally distributed variables were presented as mean ± SD and compared using independent-samples *t* tests; non-normally distributed variables were presented as median (interquartile range) and compared using the Mann–Whitney *U* test. Categorical variables were summarized as counts (percentages) and compared using the *χ*^2^ test or Fisher's exact test, as appropriate.

Univariable Cox regression analysis was used to evaluate the correlation between each variable and the 28-day and 90-day mortality. Then, the variables with a *P* value <0.05 were selected for inclusion in the multivariate Cox regression analysis and three multivariate regression analysis models were constructed (while considering the collinearity among the variables): for 28-day mortality, Model 1: unadjusted; Model 2: adjusted for TIMI 3 vs. 0–2 only; Model 3: adjusted for TIMI 3 vs. 0–2, LVEF, lactate, creatinine, and Nt-ProBNP; for 90-day mortality, Model 1: unadjusted; Model 2: adjusted for TIMI 3 vs. 0–2 only; Model 3: adjusted for TIMI 3 vs. 0–2, BMI, LVEF, lactate, creatinine, and Nt-ProBNP. Furthermore, based on the full-adjustment model, a multivariate Cox regression analysis was conducted to explore the multivariate stratified association between UA and the 28-day and 90-day mortality risks in the IABP subgroup (yes or no). Results are reported as hazard ratios (HRs) with 95% confidence intervals (CIs). ROC curves were used to evaluate the discriminatory performance of UA for 28-day and 90-day mortality, quantified by the area under the curve (AUC). Because the ROC-derived cut-offs were generated and initially evaluated within the same cohort, bootstrap internal validation with 1,000 resamples was performed to assess the stability of the exploratory cut-offs and discrimination metrics. In each bootstrap sample, the optimal UA cut-off was re-estimated using the maximal Youden index, and the AUC, sensitivity, and specificity were calculated. Bootstrap distributions were summarized as medians with 95% CI. Kaplan–Meier survival curves were constructed and compared between UA strata using the log-rank test. Restricted cubic spline (RCS) regression, with covariate adjustment, was applied to assess potential non-linear associations between UA and mortality risk; nonlinearity was assessed by comparing the model with spline terms to the model with only the linear term using a likelihood ratio test. All tests were two-sided, and *P* < 0.05 was considered statistically significant.

## 3 Results

### 3.1 Clinical characteristics stratified by serum UA

As summarized in Table 1, patients were dichotomized into a low-UA group (≤530 μmol/L) and a high-UA group (>530 μmol/L) according to the exploratory ROC-derived cut-off for 28-day mortality. Baseline UA in the overall cohort was 452.00 μmol/L (361.00–561.75 μmol/L). Under the 530 μmol/L stratification, median UA was 381.00 μmol/L (314.25–456.75 μmol/L) in the low-UA group and 638.50 μmol/L (577.75–719.00 μmol/L) in the high-UA group. Compared with the low-UA group, patients with elevated UA were younger, exhibited a lower proportion of TIMI grade 2, and had higher creatinine and triglyceride concentrations (all *P* < 0.05). Both 28-day and 90-day mortality were higher in the high-UA group than in the low-UA group (*P* = 0.002 and 0.009, respectively). No other baseline variables differed significantly between groups (*P* > 0.05).

**Table 1:** Clinical characteristics grouped by UA.

Variables	Total population	Low UA (≤530)	High UA (>530)	*P* value	Low UA (≤561)	High UA (>561)	*P* value
N	142	98	44		106	36	
Age, years	57.54 ± 11.53	59.06 ± 11.66	54.14 ± 10.57	0.018	59.09 ± 11.49	52.94 ± 10.50	0.005
Sex, *n* (%)				0.056			0.088
Male	112 (78.9)	73 (74.5)	39 (88.6)		80 (75.5)	32 (88.9)	
Female	30 (21.1)	25 (25.5)	5 (11.4)		26 (24.5)	4 (11.1)	
Smoking, *n* (%)	66 (46.5)	46 (46.9)	20 (45.5)	0.870	49 (46.2)	17 (47.2)	0.918
Hypertension, *n* (%)	53 (37.3)	37 (37.8)	16 (36.4)	0.874	38 (35.8)	15 (41.7)	0.533
Diabetes, *n* (%)	45 (31.7)	32 (32.7)	13 (29.5)	0.713	33 (31.1)	12 (33.3)	0.806
Previous MI, *n* (%)	18 (12.7)	14 (14.3)	4 (9.1)	0.390	14 (13.2)	4 (11.1)	1.000
SCAI shock state, *n* (%)				0.807			0.693
C	16 (11.3)	10 (10.2)	6 (13.6)		11 (10.4)	5 (13.9)	
D	80 (56.3)	55 (56.1)	25 (56.8)		59 (55.7)	21 (58.3)	
E	46 (32.4)	33 (33.7)	13 (29.5)		36 (34.0)	10 (27.8)	
Resuscitation before ECMO, *n* (%)	53 (37.3)	35 (35.7)	18 (40.9)	0.554	42 (39.6)	11 (30.6)	0.331
BMI, kg/m^2^	24.80 (22.67, 27.08)	24.63 (22.39, 26.45)	25.53 (22.86, 27.68)	0.107	24.49 (22.26, 26.73)	25.92 (24.01, 27.67)	0.031
SBP, mmHg	70.00 (0.00, 85.25)	70.00 (40.00, 86.00)	69.50 (0.00, 82.00)	0.403	70.00 (0.00, 86.00)	74.50 (15.00, 85.00)	0.616
DBP, mmHg	45.00 (20.00, 60.00)	45.00 (20.00, 60.00)	45.00 (0.00, 52.75)	0.153	42.00 (0.00, 59.25)	48.00 (7.50, 55.50)	0.964
HR, beats/min	76.00 (0.00, 109.25)	76.00 (19.50, 109.25)	74.00 (0.00, 109.00)	0.492	68.50 (0.00, 108.25)	83.50 (0.00, 110.00)	0.554
Diseased vessels ≥2, *n* (%)	84 (59.2)	57 (58.2)	27 (61.4)	0.720	61 (57.5)	23 (63.9)	0.504
Culprit vessels ≥2, *n* (%)	52 (36.6)	34 (34.7)	18 (40.9)	0.477	36 (34.0)	16 (44.4)	0.259
Stents implanted ≥2, *n* (%)	28 (19.7)	17 (17.3)	11 (25.0)	0.289	18 (17.0)	10 (27.8)	0.160
TIMI grade, *n* (%)				0.048			0.241
0	8 (5.6)	3 (3.1)	5 (11.4)		5 (4.7)	3 (8.3)	
1	1 (0.7)	0 (0.0)	1 (2.3)		0 (0.0)	1 (2.8)	
2	13 (9.2)	11 (11.2)	2 (4.5)		11 (10.4)	2 (5.6)	
3	120 (84.5)	84 (85.7)	36 (81.8)		90 (84.9)	30 (83.3)	
Time to revascularization, h	7.00 (0.00, 24.00)	6.50 (3.50, 16.00)	8.50 (5.00, 48.00)	0.138	6.00 (3.88, 16.75)	9.00 (5.00, 48.00)	0.080
LVEF, %	33.92 ± 9.63	34.78 ± 10.24	32.00 ± 7.91	0.113	35.01 ± 10.14	30.69 ± 7.16	0.007
Lactate, mmol/L	5.20 (4.00, 24.00)	5.00 (3.30, 7.50)	5.95 (3.43, 10.18)	0.118	5.15 (3.30, 7.75)	5.65 (3.43, 9.45)	0.374
WBC, ×10^9^/L	18.31 (14.72, 21.83)	17.25 (14.17, 21.66)	19.04 (15.97, 22.78)	0.110	17.25 (14.15, 21.66)	19.10 (16.34, 22.88)	0.038
Hemoglobin, g/L	121.00 (100.75, 135.00)	119.00 (100.75, 132.25)	126.50 (98.50, 138.50)	0.192	120.50 (101.75, 133.00)	126.50 (91.25, 138.50)	0.653
Platelet, ×10^9^/L	219.00 (169.00, 273.50)	215.50 (168.75, 265.50)	233.00 (167.00, 285.75)	0.240	215.50 (168.75, 264.25)	215.50 (167.00, 305.50)	0.099
Albumin, g/L	34.90 ± 5.07	34.92 ± 4.93	34.86 ± 5.41	0.946	35.12 ± 4.89	34.24 ± 5.58	0.369
Creatinine, μmol/L	122.00 (91.00, 168.25)	110.00 (84.75, 143.50)	153.10 (117.50, 224.75)	<0.001	111.50 (84.75, 143.25)	164.50 (128.25, 256.25)	<0.001
UA, μmol/L	452.00 (361.00, 561.75)	381.00 (314.25, 456.75)	638.50 (577.75, 719.00)	<0.001	392.50 (327.75, 477.50)	664.50 (600.75, 777.00)	<0.001
Potassium, mmol/L	4.36 (3.98, 4.66)	4.29 (3.95, 4.63)	4.46 (4.04, 5.08)	0.112	4.31 (3.95, 4.65)	4.40 (4.04, 5.09)	0.234
Sodium, mmol/L	139.00 (136.00, 141.53)	139.15 (136.60, 141.50)	138.00 (135.00, 142.53)	0.387	139.00 (136.58, 141.50)	138.50 (134.93, 142.53)	0.476
Calcium, mmol/L	2.08 ± 0.19	2.07 ± 0.18	2.09 ± 0.21	0.545	2.08 ± 0.19	2.07 ± 0.21	0.841
Glucose, mmol/L	13.22 (10.43, 18.28)	13.45 (10.43, 18.06)	12.90 (10.40, 20.26)	0.860	13.45 (10.43, 18.06)	12.90 (9.98, 20.92)	0.855
Total cholesterol, mmol/L	3.89 (3.16, 5.05)	3.79 (3.13, 5.00)	4.29 (3.17, 5.10)	0.132	3.79 (3.14, 5.00)	4.65 (3.17, 5.10)	0.100
Triglycerides, mmol/L	1.17 (0.86, 1.85)	1.09 (0.78, 1.73)	1.40 (0.96, 1.86)	0.048	1.10 (0.80, 1.79)	1.40 (0.96, 1.86)	0.145
LDL-C, mmol/L	2.36 (1.77, 3.28)	2.21 (1.73, 3.21)	2.50 (1.87, 3.51)	0.218	2.21 (1.73, 3.21)	2.53 (1.97, 3.51)	0.222
HDL-C, mmol/L	1.00 (0.78, 1.23)	1.04 (0.80, 1.26)	0.91 (0.73, 1.19)	0.148	1.02 (0.80, 1.25)	0.91 (0.73, 1.23)	0.286
Fibrinogen, g/L	3.44 (2.52, 4.77)	3.48 (2.72, 4.82)	3.36 (2.38, 4.63)	0.354	3.30 (2.55, 4.82)	3.69 (2.45, 4.63)	0.909
cTnT, ng/mL	10.00 (4.23, 10.00)	9.16 (4.26, 10.00)	10.00 (4.10, 10.00)	0.643	9.05 (4.26, 10.00)	10.00 (4.10, 10.00)	0.521
CK-MB, ng/mL	212.10 (48.79, 300.00)	182.25 (44.04, 300.00)	251.50 (65.42, 300.00)	0.568	204.25 (44.04, 300.00)	229.65 (65.42, 300.00)	0.812
Nt-ProBNP, pg/mL	4,709.50 (1,854.00, 11,167.00)	4,287.00 (1,875.75, 9,279.25)	6,868.50 (1,652.75, 12,344.25)	0.341	4,090.00 (1,778.75, 9,098.50)	7,170.00 (1,881.50, 21,521.25)	0.083
ECMO timing, *n* (%)				0.097			0.093
Before revascularization	103 (72.5)	67 (68.4)	36 (81.8)		73 (68.9)	30 (83.3)	
After revascularization	39 (27.5)	31 (31.6)	8 (18.2)		33 (31.1)	6 (16.7)	
ECMO initiation location, *n* (%)				0.874			0.625
In-hospital	102 (71.8)	70 (71.4)	32 (72.7)		75 (70.8)	27 (75.0)	
Out-of-hospital	40 (28.2)	28 (28.6)	12 (27.3)		31 (29.2)	9 (25.0)	
IABP, *n* (%)	61 (43.0)	40 (40.8)	21 (47.7)	0.442	43 (40.6)	18 (50.0)	0.323
ECMO duration, days	9.00 (6.00, 12.00)	9.00 (6.00, 12.00)	8.00 (3.25, 11.00)	0.113	8.50 (6.00, 12.00)	9.00 (3.25, 11.75)	0.670
Mechanical ventilation duration, days	1.50 (1.00, 6.00)	2.00 (1.00, 6.25)	1.00 (1.00, 5.00)	0.435	2.00 (1.00, 6.25)	1.00 (0.25, 4.75)	0.294
IABP duration, days	0.00 (0.00, 9.00)	0.00 (0.00, 8.25)	0.00 (0.00, 9.00)	0.454	0.00 (0.00, 8.25)	1.00 (0.00, 11.25)	0.281
CRRT duration, days	0.00 (0.00, 0.00)	0.00 (0.00, 0.00)	0.00 (0.00, 1.00)	0.091	0.00 (0.00, 0.00)	0.00 (0.00, 1.75)	0.032
28-day mortality, *n* (%)				0.002			0.001
Yes	48 (33.8)	25 (25.5)	23 (52.3)		28 (26.4)	20 (55.6)	
No	94 (66.2)	73 (74.5)	21 (47.7)		78 (73.6)	16 (44.4)	
90-day mortality, *n* (%)				0.009			0.004
Yes	58 (40.8)	33 (33.7)	25 (56.8)		36 (43.3)	22 (61.1)	
No	84 (59.2)	65 (66.3)	19 (43.2)		70 (66.0)	14 (38.9)	

UA, uric acid; BMI, body mass index; SBP, systolic blood pressure; DBP, diastolic blood pressure; HR, heart rate; MI, myocardial infarction; SCAI, Society for Cardiovascular Angiography and Interventions; ECMO, extracorporeal membrane oxygenation; TIMI, Thrombolysis in Myocardial Infarction; LVEF, left ventricular ejection fraction; WBC, white blood cell; cTnT, cardiac troponin T; CK-MB, creatine kinase-MB; Nt-ProBNP, N-terminal pro-brain natriuretic peptide; IABP, intra-aortic balloon pump; CRRT, continuous renal replacement therapy.

Using the exploratory ROC-derived cut-off for 90-day mortality (561 μmol/L), patients were reclassified into a low-UA group (≤561 μmol/L) and a high-UA group (>561 μmol/L). The median UA values were 392.50 μmol/L (327.75–477.50 μmol/L) in the low-UA group and 664.50 μmol/L (600.75–777.00 μmol/L) in the high-UA group. Under this stratification, the high-UA group remained younger but also displayed lower LVEF, accompanied by higher BMI, WBC count, creatinine level, and longer CRRT duration (all *P* < 0.05). Consistently, 28-day and 90-day mortality were increased among patients with UA > 561 μmol/L (*P* = 0.001 and 0.004, respectively), whereas the remaining clinical variables were comparable between groups (*P* > 0.05).

### 3.2 Clinical characteristics according to 28-day and 90-day mortality

Table 2 presented clinical features stratified by survival status. When grouped by 28-day outcome (death, *n* = 48; survival, *n* = 94), patients who died within 28 days demonstrated lower LVEF and a reduced prevalence of TIMI grade 3, while exhibiting higher WBC count, creatinine, and NT-proBNP, together with prolonged mechanical ventilation, longer CRRT duration, and higher CRRT intensity/level (all *P* < 0.05).

**Table 2:** Clinical characteristics grouped by 28-day and 90-day mortality.

Variables	28-day mortality			90-day mortality		
	No	Yes	*P* value	No	Yes	*P* value
N	94	48		84	58	
Age, years	56.97 ± 11.70	58.65 ± 11.21	0.414	56.18 ± 11.59	59.50 ± 11.25	0.092
Sex, *n* (%)			0.352			0.088
Male	72 (76.6)	40 (83.3)		64 (76.2)	48 (82.8)	
Female	22 (23.4)	8 (16.7)		20 (23.8)	10 (17.2)	
Smoking, *n* (%)	43 (45.7)	23 (47.9)	0.806	39 (46.4)	27 (46.6)	0.988
Hypertension, *n* (%)	31 (33.0)	22 (45.8)	0.134	28 (33.3)	25 (43.1)	0.237
Diabetes, *n* (%)	30 (31.9)	15 (31.3)	0.936	27 (32.1)	18 (31.0)	0.889
Previous MI, *n* (%)	9 (9.6)	9 (18.8)	0.120	8 (10.6)	10 (17.2)	0.174
SCAI shock state, *n* (%)			0.781			0.726
C	11 (11.7)	5 (10.4)		10 (11.9)	6 (10.3)	
D	51 (54.3)	29 (60.4)		45 (53.6)	35 (60.3)	
E	32 (34.0)	14 (29.2)		29 (34.5)	17 (29.3)	
Resuscitation before ECMO, *n* (%)	36 (38.3)	17 (35.4)	0.737	35 (41.7)	18 (31.0)	0.918
BMI, kg/m^2^	25.06 (22.81, 27.55)	24.22 (22.62, 27.00)	0.301	25.31 (22.86, 27.62)	24.22 (22.39, 26.26)	0.049
SBP, mmHg	72.50 (0.00, 86.00)	68.00 (0.00, 80.00)	0.301	71.00 (0.00, 86.00)	70.00 (37.50, 83.50)	0.910
DBP, mmHg	45.50 (0.00, 60.00)	45.00 (0.00, 60.00)	0.568	41.00 (0.00, 57.50)	47.00 (22.50, 59.00)	0.422
HR, beats/min	68.50 (0.00, 103.00)	88.50 (0.00, 110.00)	0.467	65.50 (0.00, 102.00)	90.00 (22.50, 110.00)	0.095
Diseased vessels ≥2, *n* (%)	55 (58.5)	29 (60.4)	0.827	48 (57.1)	36 (62.1)	0.557
Culprit vessels ≥2, *n* (%)	31 (33.0)	21 (43.8)	0.208	27 (32.1)	25 (43.1)	0.183
Stents implanted ≥2, *n* (%)	17 (18.5)	11 (22.9)	0.494	15 (17.9)	13 (22.4)	0.502
TIMI grade, *n* (%)			0.001			0.005
0	3 (3.2)	5 (10.4)		2 (2.4)	6 (10.3)	
1	0 (0.0)	1 (2.1)		0 (0.0)	1 (1.7)	
2	4 (4.3)	9 (18.8)		4 (4.8)	9 (15.5)	
3	87 (92.6)	33 (68.8)		78 (92.9)	42 (72.4)	
Time to revascularization, h	7.00 (4.00, 16.00)	7.00 (4.00, 48.00)	0.423	6.50 (4.00, 19.75)	7.25 (4.00, 40.50)	0.682
LVEF, %	36.21 ± 9.51	29.42 ± 8.27	<0.001	37.06 ± 9.32	29.36 ± 8.21	<0.001
Lactate, mmol/L	5.00 (3.28, 7.50)	6.35 (3.43, 9.68)	0.071	4.80 (3.05, 7.48)	6.10 (3.65, 9.43)	0.018
WBC, ×10^9^/L	16.99 (13.87, 21.66)	19.35 (15.97, 22.62)	0.025	16.67 (12.90, 22.10)	19.00 (15.83, 21.65)	0.020
Hemoglobin, g/L	120.50 (99.50, 133.50)	122.00 (101.50, 135.00)	0.988	123.00 (100.50, 135.00)	118.00 (100.75, 134.25)	0.554
Platelet, ×10^9^/L	213.50 (165.75, 273.50)	228.00 (176.25, 278.50)	0.600	212.50 (164.25, 276.50)	224.50 (186.50, 264.25)	0.706
Albumin, g/L	35.31 ± 5.12	34.09 ± 4.92	0.174	35.38 ± 5.24	34.20 ± 4.77	0.173
Creatinine, μmol/L	113.00 (83.00, 145.00)	149.10 (109.75, 202.75)	<0.001	109.00 (80.00, 142.75)	143.00 (111.25, 202.25)	<0.001
Potassium, mmol/L	4.34 (3.94, 4.64)	4.40 (4.03, 4.80)	0.309	4.28 (3.92, 4.63)	4.41 (4.05, 4.81)	0.159
Sodium, mmol/L	139.00 (136.00, 141.28)	138.75 (135.68, 142.53)	0.682	139.25 (136.05, 141.43)	138.75 (135.60, 142.18)	0.495
Calcium, mmol/L	2.08 ± 0.20	2.07 ± 0.17	0.667	2.09 ± 0.21	2.07 ± 0.17	0.550
Glucose, mmol/L	13.22 (10.15, 18.06)	13.25 (11.23, 20.43)	0.277	13.22 (10.16, 18.13)	13.25 (10.88, 20.42)	0.407
Total cholesterol, mmol/L	3.90 (3.20, 5.14)	3.86 (3.09, 4.77)	0.725	3.92 (3.17, 5.13)	3.83 (3.14, 4.83)	0.648
Triglycerides, mmol/L	1.16 (0.85, 1.91)	1.21 (0.86, 1.65)	0.481	1.23 (0.87, 1.92)	1.14 (0.83, 1.62)	0.190
LDL-C, mmol/L	2.42 (1.85, 3.55)	2.33 (1.65, 3.04)	0.150	2.42 (1.86, 3.52)	2.33 (1.66, 3.06)	0.170
HDL-C, mmol/L	0.98 (0.78, 1.18)	1.06 (0.76, 1.29)	0.349	0.98 (0.77, 1.17)	1.06 (0.78, 1.30)	0.207
Fibrinogen, g/L	3.24 (2.43, 4.83)	3.67 (2.73, 4.51)	0.652	3.21 (2.39, 4.81)	3.67 (2.76, 4.62)	0.374
cTnT, ng/mL	8.62 (4.30, 10.00)	10.00 (3.84, 10.00)	0.366	8.28 (4.42, 10.00)	10.00 (3.95, 10.00)	0.311
CK-MB, ng/mL	182.25 (28.68, 300.00)	278.60 (106.63, 300.00)	0.129	177.55 (27.75, 300.00)	278.60 (108.88, 300.00)	0.077
Nt-ProBNP, pg/mL	3,402.50 (1,357.25, 8,052.75)	6,995.50 (2,373.63, 15,470.00)	0.008	3,039.00 (1,264.50, 7,456.50)	6,995.50 (2,413.88, 20,622.75)	0.001
ECMO timing, *n* (%)			0.206			0.262
Before revascularization	65 (69.1)	38 (79.2)		58 (69.0)	45 (77.6)	
After revascularization	29 (30.9)	10 (20.8)		26 (31.0)	13 (22.4)	
ECMO initiation location, *n* (%)			0.165			0.375
In-hospital	64 (68.1)	38 (79.2)		58 (69.0)	44 (75.9)	
Out-of-hospital	30 (31.9)	10 (20.8)		26 (31.0)	14 (24.1)	
IABP, *n* (%)	37 (39.4)	24 (50.0)	0.226	30 (35.7)	31 (53.4)	0.036
ECMO duration, days	9.00 (6.00, 12.00)	8.00 (4.00, 11.75)	0.409	8.00 (6.00, 11.75)	9.00 (5.50, 12.25)	0.988
Mechanical ventilation duration, days	1.00 (0.00, 4.25)	4.50 (1.00, 8.75)	0.001	1.00 (0.00, 4.00)	4.00 (1.00, 8.25)	0.003
IABP duration, days	0.00 (0.00, 9.25)	0.00 (0.00, 6.75)	0.861	0.00 (0.00, 9.00)	1.00 (0.00, 8.25)	0.210
CRRT duration, days	0.00 (0.00, 0.00)	0.00 (0.00, 2.00)	<0.001	0.00 (0.00, 0.00)	0.00 (0.00, 2.00)	<0.001
UA, μmol/L	433.50 (345.25, 525.25)	516.50 (380.75, 608.75)	0.011	433.50 (327.75, 525.75)	488.50 (380.00, 598.25)	0.015
Standardized UA	−0.24 (−0.72, 0.27)	0.22 (−0.53, 0.73)	0.011	−0.24 (−0.82, 0.27)	0.07 (−0.53, 0.68)	0.015
UA categorical variable, *n* (%)			0.002			0.009
Low UA (≤530)	73 (77.7)	25 (52.1)		65 (77.4)	33 (56.9)	
High UA (>530)	21 (22.3)	23 (47.9)		19 (22.6)	25 (43.1)	
UA categorical variable, *n* (%)			0.001			0.004
Low UA (≤561)	78 (83.0)	28 (58.3)		70 (83.3)	36 (62.1)	
High UA (>561)	16 (17.0)	20 (41.7)		14 (21.3)	22 (37.9)	

UA, uric acid; BMI, body mass index; SBP, systolic blood pressure; DBP, diastolic blood pressure; HR, heart rate; MI, myocardial infarction; SCAI, Society for Cardiovascular Angiography and Interventions; ECMO, extracorporeal membrane oxygenation; TIMI, Thrombolysis in Myocardial Infarction; LVEF, left ventricular ejection fraction; WBC, white blood cell; cTnT, cardiac troponin T; CK-MB, creatine kinase-MB; Nt-ProBNP, N-terminal pro-brain natriuretic peptide; IABP, intra-aortic balloon pump; CRRT, continuous renal replacement therapy.

A broadly concordant pattern was observed for 90-day mortality: patients who died by 90 days showed lower LVEF and higher lactate, WBC, creatinine, NT-proBNP, increased IABP utilization, longer durations of mechanical ventilation and CRRT, and higher UA levels (all *P* < 0.05). No statistically significant differences were detected for the remaining variables (*P* > 0.05).

### 3.3 Univariable Cox regression for 28-day and 90-day mortality

As shown in Table 3, univariable Cox regression identified TIMI grade, LVEF, lactate, creatinine, NT-proBNP, and UA as significant correlates of both 28-day and 90-day mortality (all *P* < 0.05). Quantitatively, each 1 μmol/L increment in UA was associated with a 0.2% increase in the hazard of death at 28 days and 90 days (HR: 1.002, 95% CI: 1.001–1.004, *P* = 0.003; HR: 1.002, 95% CI: 1.001–1.003, *P* = 0.001). When scaled per 1-SD increase, UA was associated with a 47.1% and 47.8% elevation in the hazard of 28-day and 90-day mortality, respectively (HR: 1.471, 95% CI: 1.137–1.905, *P* = 0.003; HR: 1.478, 95% CI: 1.164–1.877, *P* = 0.001).

**Table 3:** Univariate Cox regression analysis for 28-day and 90-day mortality.

Variables	28-day mortality			90-day mortality		
	HR	95% CI	*P* value	HR	95% CI	*P* value
Age	1.013	0.988–1.038	0.324	1.020	0.997–1.040	0.087
Sex						
Male	1.401	0.656–2.994	0.384	1.363	0.690–2.694	0.373
Female	Ref			Ref		
Smoking	1.068	0.606–1.881	0.820	1.011	0.604–1.695	0.695
Hypertension	1.549	0.877–2.734	0.131	1.393	0.828–2.344	0.211
Diabetes	0.960	0.521–1.767	0.896	0.948	0.544–1.654	0.852
Previous MI	1.927	0.932–3.986	0.077	1.790	0.904–3.543	0.095
SCAI Shock state						
C	Ref			Ref		
D	1.237	0.479–3.196	0.661	1.249	0.525–2.971	0.614
E	1.010	0.364–2.804	0.985	1.016	0.400–2.576	0.974
Resuscitation before ECMO	0.931	0.515–1.681	0.812	0.751	0.431–1.311	0.314
BMI, kg/m^2^	0.941	0.862–1.027	0.173	0.917	0.845–0.995	0.038
SBP, mmHg	0.998	0.990–1.006	0.626	1.001	0.994–1.008	0.788
DBP, mmHg	0.998	0.987–1.009	0.742	1.003	0.993–1.014	0.561
HR, beats/min	1.002	0.996–1.007	0.587	1.003	0.998–1.009	0.197
Diseased vessels ≥2	1.014	0.568–1.808	0.963	1.097	0.645–1.865	0.733
Culprit vessels ≥2	1.364	0.771–2.413	0.286	1.350	0.802–2.270	0.258
Stents implanted ≥2	1.164	0.594–2.281	0.659	1.136	0.613–2.107	0.685
TIMI grade						
0	Ref			Ref		
1	1.819	0.208–15.864	0.588	1.713	0.202–14.525	0.621
2	0.917	0.307–2.743	0.877	0.774	0.275–2.177	0.627
3	0.241	0.094–0.619	0.003	0.248	0.105–0.585	0.001
Time to revascularization	1.001	0.999–1.003	0.344	1.001	0.998–1.003	0.593
LVEF	0.932	0.900–0.964	<0.001	0.927	0.898–0.957	<0.001
Lactate	1.083	1.013–1.158	0.019	1.091	1.027–1.160	0.005
WBC	1.034	0.997–1.072	0.073	1.032	0.998–1.067	0.069
Hemoglobin	1.000	0.988–1.012	0.986	0.998	0.987–1.008	0.690
Platelet	0.999	0.997–1.002	0.620	0.999	0.997–1.002	0.476
Albumin	0.966	0.917–1.018	0.201	0.967	0.921–1.015	0.173
Creatinine	1.003	1.001–1.005	<0.001	1.003	1.002–1.005	<0.001
Potassium	1.205	0.831–1.748	0.326	1.252	0.897–1.746	0.186
Sodium	0.976	0.912–1.004	0.477	0.961	0.902–1.024	0.218
Calcium	0.748	0.176–3.187	0.695	0.700	0.188–2.606	0.595
Glucose	1.036	0.992–1.083	0.109	1.032	0.991–1.075	0.131
Total cholesterol	1.012	0.821–1.248	0.908	1.003	0.828–1.214	0.979
Triglycerides	0.814	0.565–1.171	0.267	0.791	0.565–1.107	0.172
LDL-C	0.832	0.637–1.085	0.175	0.859	0.677–1.089	0.210
HDL-C	1.411	0.782–2.546	0.253	1.649	1.000–2.718	0.050
Fibrinogen	1.043	0.878–1.240	0.630	1.069	0.916–1.248	0.396
cTnT	1.035	0.949–1.127	0.439	1.032	0.955–1.116	0.427
CK-MB	1.002	0.999–1.004	0.154	1.002	1.000–1.004	0.092
Nt-ProBNP	1.000	1.000–1.000	0.010	1.000	1.000–1.000	<0.001
ECMO timing						
Before revascularization	Ref			Ref		
After revascularization	0.654	0.326–1.312	0.231	0.711	0.384–1.318	0.279
ECMO initiation location						
In-hospital	Ref			Ref		
Out-of-hospital	0.624	0.311–1.252	0.184	0.745	0.408–1.359	0.336
IABP	1.382	0.785–2.435	0.262	1.623	0.968–2.720	0.066
ECMO duration	0.998	0.961–1.037	0.921	1.006	0.976–1.038	0.681
Mechanical ventilation duration	1.026	0.996–1.058	0.092	1.027	0.999–1.056	0.062
IABP duration	0.986	0.951–1.022	0.427	1.002	0.973–1.031	0.919
CRRT duration	1.020	0.983–1.058	0.301	1.025	0.993–1.058	0.132
UA	1.002	1.001–1.004	0.003	1.002	1.001–1.003	0.001
Standardized UA	1.471	1.137–1.905	0.003	1.478	1.164–1.877	0.001
UA categorical variable						
Low UA (≤530)	Ref			Ref		
High UA (>530)	2.592	1.470–4.570	0.001	2.194	1.304–3.693	0.003
UA categorical variable						
Low UA (≤561)	Ref			Ref		
High UA (>561)	2.632	1.481–4.676	0.001	2.341	1.375–3.984	0.002

UA, uric acid; BMI, body mass index; SBP, systolic blood pressure; DBP, diastolic blood pressure; HR, heart rate; MI, myocardial infarction; SCAI, Society for Cardiovascular Angiography and Interventions; ECMO, extracorporeal membrane oxygenation; TIMI, Thrombolysis in Myocardial Infarction; LVEF, left ventricular ejection fraction; WBC, white blood cell; cTnT, cardiac troponin T; CK-MB, creatine kinase-MB; Nt-ProBNP, N-terminal pro-brain natriuretic peptide; IABP, intra-aortic balloon pump; CRRT, continuous renal replacement therapy; Ref, reference category.

Using 530 μmol/L as the threshold, high UA conferred a 2.592-fold higher risk of 28-day mortality (95% CI: 1.470–4.570, *P* = 0.001) and a 2.194-fold higher risk of 90-day mortality (95% CI: 1.304–3.693, *P* = 0.003) relative to low UA. With 561 μmol/L as the cut-off, the excess risk increased to 2.632-fold for 28-day mortality (95% CI: 1.481–4.676, *P* = 0.001) and 2.341-fold for 90-day mortality (95% CI: 1.375–3.984, *P* = 0.002).

### 3.4 Association between UA and mortality risk at 28 and 90 days

The independent relationship between UA and short- and intermediate-term mortality was further corroborated in multivariable Cox models (Table 4). After adjustment for TIMI 3 vs. TIMI 0–2 and prespecified clinical covariates, each 1 μmol/L increase in UA remained associated with a higher hazard of 28-day mortality (HR: 1.002, 95% CI: 1.000–1.003, *P* = 0.038), and each 1-SD increase corresponded to a 37.9% increase in risk (HR: 1.379, 95% CI: 1.019–1.867, *P* = 0.038). Consistently, UA > 530 μmol/L was associated with a 2.499-fold higher risk of 28-day death compared with UA ≤ 530 μmol/L (95% CI: 1.335–4.677, *P* = 0.004).

**Table 4:** Association between UA and mortality.

Variables	Model 1			Model 2			Model 3		
	HR	95% CI	*P* value	HR	95% CI	*P* value	HR	95% CI	*P* value
28-day mortality									
UA as continuous variable									
UA	1.002	1.001–1.004	0.003	1.002	1.001–1.004	0.002	1.002	1.000–1.003	0.038
Standardized UA	1.471	1.137–1.905	0.003	1.495	1.156–1.934	0.002	1.379	1.019–1.867	0.038
UA as categorical variable (530)									
Low UA group	Ref			Ref			Ref		
High UA group	2.592	1.470–4.570	0.001	3.004	1.684–5.357	<0.001	2.499	1.335–4.677	0.004
90-day mortality									
UA as continuous variable									
UA	1.002	1.001–1.003	0.001	1.002	1.001–1.004	<0.001	1.002	1.001–1.004	0.005
Standardized UA	1.478	1.164–1.877	0.001	1.504	1.184–1.910	<0.001	1.494	1.126–1.982	0.005
UA as categorical variable (561)									
Low UA group	Ref			Ref			Ref		
High UA group	2.341	1.375–3.984	0.002	2.596	1.514–4.450	<0.001	2.262	1.222–4.188	0.009

For 28-day mortality, Model 1: unadjusted; Model 2: adjusted for TIMI 3 vs. 0–2 only; Model 3: adjusted for TIMI 3 vs. 0–2, LVEF, lactate, creatinine, and Nt-ProBNP.

For 90-day mortality, Model 1: unadjusted; Model 2: adjusted for TIMI 3 vs. 0–2 only; Model 3: adjusted for TIMI 3 vs. 0–2, BMI, LVEF, lactate, creatinine, and Nt-ProBNP.

UA, uric acid; BMI, body mass index; TIMI, Thrombolysis in Myocardial Infarction; LVEF, left ventricular ejection fraction; Nt-ProBNP, N-terminal pro-brain natriuretic peptide; Ref, reference category; HR, hazard ratio; CI, confidence interval.

For 90-day mortality, UA was likewise associated with outcome: each 1 μmol/L increase was linked to a higher hazard (HR: 1.002, 95% CI: 1.001–1.004, *P* = 0.005), while each 1-SD increase corresponded to a 49.4% elevation in 90-day mortality risk (HR: 1.494, 95% CI: 1.126–1.982, *P* = 0.005). Patients with UA > 561 μmol/L had a 2.262-fold higher risk of 90-day mortality than those with UA ≤ 561 μmol/L (95% CI: 1.222–4.188, *P* = 0.009).

Considering the significance of IABP in VA-ECMO, an additional subgroup analysis based on IABP was conducted (Supplementary Table S1). The results showed that in the subgroup without IABP, for every 1 SD increase in UA, the 28-day and 90-day mortality risks increased by 49.5% and 58.2% respectively (*P* < 0.05). Moreover, the 28-day and 90-day mortality risks in the high UA group were 2.403 times and 3.003 times those of the low UA group, respectively (*P* < 0.05). However, in the subgroup with IABP, only the high UA group had a higher 28-day mortality risk (*P* = 0.016), while the others showed no statistical significance.

### 3.5 Visualization of the prognostic performance of UA

**Figure 1:**
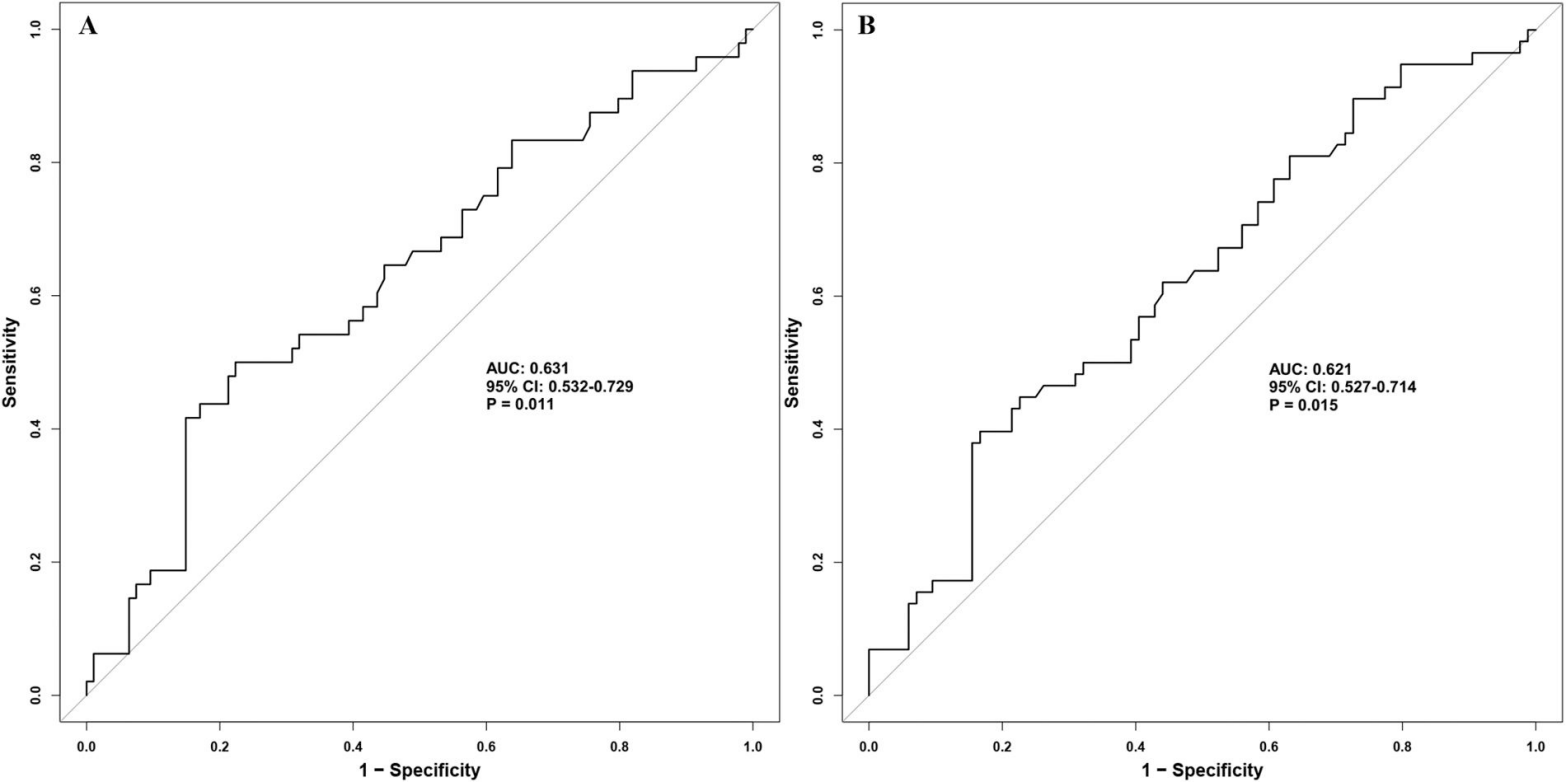
ROC curves for UA in predicting 28-day **(A)** and 90-day mortality **(B)** UA, uric acid; ROC, receiver operating characteristic; AUC, area under the curve; CI, confidence interval.

As depicted in Figure 1, ROC analysis was performed as an exploratory assessment of UA discrimination for 28-day and 90-day mortality. In the original cohort, the apparent AUCs were 0.631 (95% CI: 0.532–0.728) and 0.621 (95% CI: 0.523–0.715), respectively, with apparent Youden-derived cut-offs of 530 μmol/L and 561 μmol/L. Bootstrap internal validation showed modest discrimination and sampling variability in the re-estimated cut-offs. The bootstrap median cut-off was 534 μmol/L (371–586 μmol/L) for 28-day mortality and 530 μmol/L (300–571 μmol/L) for 90-day mortality (Supplementary Table S2).

**Figure 2:**
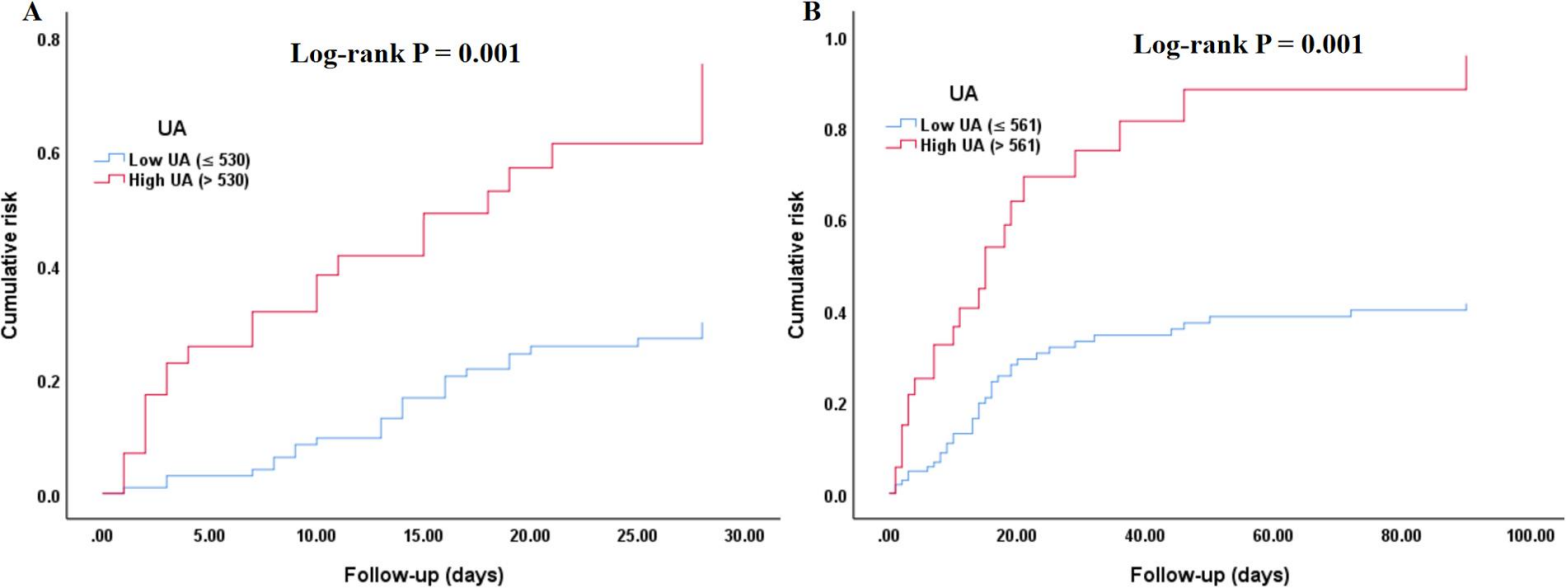
Kaplan–Meier survival curves showing the differences in cumulative risks of 28-day mortality **(A)** and 90-day mortality **(B)** among different UA groups. UA, uric acid.

Kaplan–Meier curves further demonstrated pronounced divergence in cumulative mortality risk between UA strata (Figure 2). For the 28-day endpoint, patients with UA > 530 μmol/L exhibited higher cumulative mortality than those with UA ≤ 530 μmol/L (log-rank *P* = 0.001; Figure 2A). Similarly, for the 90-day endpoint, UA >561 μmol/L was associated with lower survival rate compared with UA ≤ 561 μmol/L (log-rank *P* = 0.001; Figure 2B).

**Figure 3:**
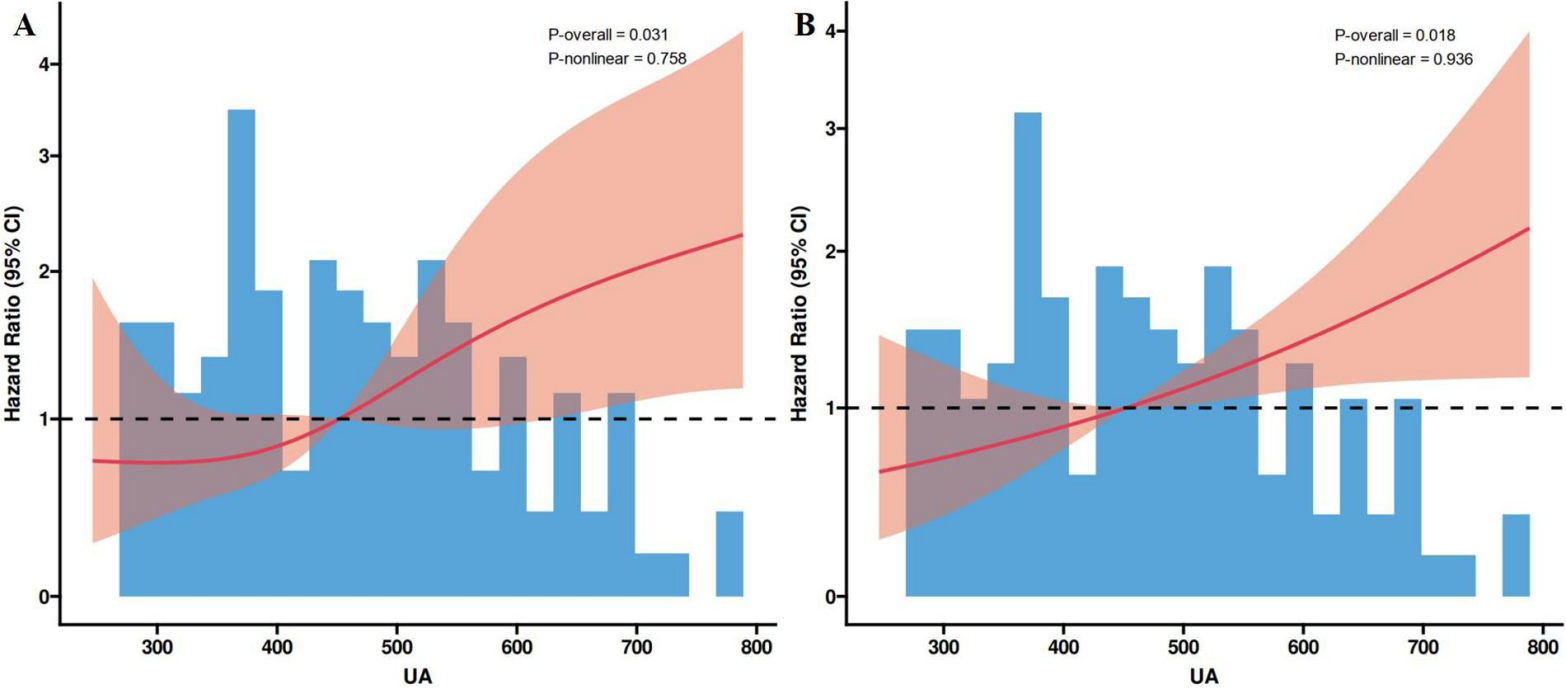
RCS plots showing the correlation between UA and 28-day mortality **(A)** and 90-day mortality **(B)** UA, uric acid; RCS, restricted cubic spline.

RCS analyses supported a statistically linear association between UA and mortality risk at both 28 days (*P* = 0.031; *P* for non-linearity = 0.758) and 90 days (*P* = 0.018; *P* for non-linearity = 0.936), with no evidence of a non-linear relationship (Figure 3).

## 4 Discussion

Leveraging real-world clinical data, this study applied a suite of complementary statistical approaches to interrogate, from multiple perspectives, the relationship between UA and 28-day as well as 90-day mortality after VA-ECMO support in patients with AMI-CS. After adjustment for prespecified confounders, elevated UA remained associated with increased risks of death at both time points. ROC analyses suggested only modest apparent discrimination. Kaplan–Meier curves showed separation across exploratory UA strata, and RCS modeling was compatible with a positive linear association between UA and mortality risk. These findings suggest that UA may provide prognostic information in this cohort, but external validation is needed.

UA, the end product of purine metabolism, exerts antioxidant effects *in vivo* and has been implicated in neuroprotection; however, its role in cardiovascular disease has remained contentious. In recent years, accumulating evidence has increasingly supported an association between higher UA levels and cardiovascular burden as well as adverse outcomes. In a large, contemporary cohort of 5,888 patients with successfully revascularized AMI, Kim et al. (9) showed that higher on-admission serum UA was associated with increased long-term all-cause mortality and improved risk discrimination when added to traditional prognostic factors. A cohort analysis (16) including 21,386 participants further demonstrated that UA is an independent risk factor for both incident HF and fatal HF: participants with UA < 5.34 mg/dL exhibited a 2.5% incidence of all-cause HF compared with 4.8% among those above this threshold, and the incidence of fatal HF was 1.6% for UA < 4.89 mg/dL vs. 3.3% for higher levels. These findings suggest that increasing UA may be associated with HF onset and prognosis. Consistently, a retrospective cohort study (17) of 757 patients with AMI and HF with preserved ejection fraction showed that higher UA was independently associated with long-term all-cause mortality; after a median follow-up of 4.8 years, mortality in the high-UA group (32.9%) was higher than that in the normouricemic group (15.5%). Moreover, a meta-analysis (18) encompassing 40 cohort studies and 105,609 patients with acute coronary syndrome (ACS) quantitatively consolidated the prognostic relevance of UA, revealing increased risks of all-cause mortality, cardiovascular mortality, and HF among patients with elevated UA. Notably, that meta-analysis also suggested a non-linear dose–response relationship between UA and mortality, raising the possibility that UA may not merely be a surrogate marker but could also contribute directly to pathogenesis. Collectively, these studies support the notion that UA can be considered an independent risk factor for adverse prognosis in AMI, particularly among patients complicated by HF. In addition, Miao et al. (19) reported a positive relationship between UA and adverse events in HF, with each 1 mg/dL increase in UA associated with a 4% rise in all-cause mortality and a 9% increase in the composite endpoint of death or cardiac events. Deis et al. (20) further observed that, in advanced HF, elevated UA correlated with more profound hemodynamic impairment and predicted long-term all-cause mortality as well as composite outcomes.

Compared with previous studies, our current research made some additional contributions in several respects. First, we focused on a specific and clinically heterogeneous cohort of patients with AMI-CS receiving VA-ECMO support. Second, we incorporated multiple conventional cardiovascular risk factors and key clinical variables, constructing multivariable models to mitigate confounding bias. Third, we examined the dose-response pattern between UA and both 28-day and 90-day mortality. Fourth, we we examined metabolic derangements in the context of mechanical circulatory support, highlighting that once hemodynamics are partially restored, metabolic indicators may retain prognostic relevance.

Despite these observations, the mechanistic pathways linking UA to short- and mid-term mortality in this critically ill cohort remain incompletely defined. Existing evidence suggests that elevated UA may contribute to cardiovascular injury through convergent mechanisms, including induction of oxidative stress, activation of inflammatory cascades, promotion of endothelial dysfunction, suppression of nitric oxide bioavailability, and activation of the RAAS (21–23). UA has also been reported to upregulate platelet-derived growth factor, thereby facilitating vascular smooth muscle cell proliferation and accelerating atherosclerotic progression (24). In parallel, hyperuricemia may precipitate or exacerbate metabolic syndrome (25), which is linked to subclinical cardiovascular injury (6). In AMI-CS supported by VA-ECMO, however, elevated UA may also reflect systemic hypoperfusion, oxygen debt, cellular injury, and impaired renal clearance rather than UA-specific biological effects alone. Of particular note, a UK Biobank analysis (26) of nearly 470,000 individuals suggested that the association between higher UA and sudden cardiac death may be mediated predominantly through renal injury, implicating kidney dysfunction as a critical node in UA-related pathogenic circuitry. In our cohort, patients in the high-UA group exhibited higher serum creatinine and longer durations of CRRT, lending clinical support to this hypothesis. Nevertheless, definitive mechanistic inference will require further experimental interrogation in cellular and animal models.

Several limitations warrant consideration. First, as a single-center retrospective analysis with a limited sample size, selection bias cannot be excluded, and external generalizability should be approached with caution. Second, as an observational retrospective cohort without genetic association analyses, our study cannot establish causality between UA and mortality risk. Third, UA was measured only once at baseline; the absence of longitudinal measurements precluded assessment of sustained exposure and temporal trajectories of UA in relation to 28-day and 90-day mortality. Fourth, although patients with documented high-purine load or purine-containing supplementation before baseline UA assessment were excluded and acute nutritional support followed institutional protocols, prehospital dietary purine intake and undocumented purine exposure could not be precisely quantified. Fifth, residual confounding by end-organ perfusion and oxygen delivery remains possible. In AMI-CS, cellular injury and elevated UA may partly reflect systemic hypoperfusion, oxygen debt, and renal dysfunction. Although baseline lactate and creatinine were included as clinically relevant surrogates, single baseline measurements cannot fully capture dynamic lactate clearance, arterial oxygen content, ECMO flow adequacy, microcirculatory perfusion, or the evolving balance between oxygen delivery and metabolic demand. Sixth, although TIMI flow was dichotomized and parsimonious models were used to reduce the degrees of freedom, the number of outcome events remained limited; therefore, model overfitting cannot be completely excluded, and the findings require validation in larger external cohorts. Seventh, available pre-baseline medication records were reviewed, and no urate-lowering therapy was documented before baseline UA measurement. However, other medications that may indirectly influence UA, such as diuretics, sodium-glucose cotransporter 2 inhibitors, or losartan, were not uniformly captured in the structured dataset; undocumented medication exposure could not be entirely excluded. Finally, although bootstrap internal validation was performed, the ROC-derived UA cut-offs were generated from a single-center cohort and remain exploratory. These thresholds should not be interpreted as definitive clinical decision limits, and external validation in larger independent cohorts is required before clinical application.

## 5 Conclusions

This study showed that higher UA levels were associated with increased 28-day and 90-day mortality among patients with AMI-CS receiving VA-ECMO. RCS analyses suggested a positive linear association between UA and mortality risk. However, because this was a single-center, retrospective, observational cohort study, these findings establish association rather than causality and should be regarded as hypothesis-generating. Further large-scale, prospective, and externally validated studies are needed to determine whether UA provides incremental prognostic value beyond established clinical risk factors and whether it can be incorporated into clinical risk assessment.

## Data Availability

The original contributions presented in the study are included in the article/Supplementary Material, further inquiries can be directed to the corresponding author.
