# Supplementary material for: A linear association between serum uric acid and 28-day and 90-day mortality after veno-arterial extracorporeal membrane oxygenation in patients with acute myocardial infarction complicated by cardiogenic shock
Source: Front Cardiovasc Med. 2026 Jul 27;13:1839659. doi: 10.3389/fcvm.2026.1839659 (PMC13453733; doi:10.3389/fcvm.2026.1839659)
Supplement: Supplementary file 1 [file Table1.pdf]

Supplementary Table 1. Exploratory subgroup analysis of the association between serum UA and mortality according to concomitant IABP support

Endpoints	IABP status	N/events	UA exposure	HR (95% CI)	P value
28-day mortality	No IABP	81/24	Per 1-SD increase in UA	1.495 (1.093-2.045)	0.012
28-day mortality	ECMO + IABP	61/24	Per 1-SD increase in UA	1.393 (0.885-2.194)	0.152
28-day mortality	No IABP	81/24	High UA > 530 μmol/L	2.403 (1.066-5.421)	0.035
28-day mortality	ECMO + IABP	61/24	High UA > 530 μmol/L	2.689 (1.203-6.006)	0.016
90-day mortality	No IABP	81/27	Per 1-SD increase in UA	1.582 (1.179-2.123)	0.002
90-day mortality	ECMO + IABP	61/31	Per 1-SD increase in UA	1.300 (0.873-1.935)	0.196
90-day mortality	No IABP	81/27	High UA > 561 μmol/L	3.003 (1.370-6.586)	0.006
90-day mortality	ECMO + IABP	61/31	High UA > 561 μmol/L	1.710 (0.829-3.526)	0.146

UA, uric acid; IABP, intra-aortic balloon pump; ECMO, extracorporeal membrane oxygenation; HR, hazard ratio; CI, confidence interval.

Supplementary Table 2. Bootstrap stability assessment of ROC-derived UA cut-offs

Endpoints	Original AUC (bootstrap 95% CI)	Original cut-off (μmol/L)	Original sensitivity	Original specificity	Bootstrap AUC, median (95% interval)	Bootstrap cut-off, median (95% interval)
28-day mortality	0.631 (0.532-0.728)	530	0.500	0.777	0.631 (0.532-0.728)	534 (371-586)
90-day mortality	0.621 (0.523-0.715)	561	0.397	0.833	0.621 (0.523-0.715)	530 (300-571)

AUC, area under the curve; ROC, receiver operating characteristic; UA, uric acid. Bootstrap resampling was performed with 1,000 repetitions. Cut-offs were re-estimated in each bootstrap sample using the maximal Youden index; therefore, these values should be regarded as exploratory stability estimates rather than externally validated clinical thresholds.
